# Immunogenic cell death: The cornerstone of oncolytic viro-immunotherapy

**DOI:** 10.3389/fimmu.2022.1038226

**Published:** 2023-01-23

**Authors:** Lalitha Palanivelu, Ching-Hsuan Liu, Liang-Tzung Lin

**Affiliations:** ^1^ International Ph.D. Program in Medicine, College of Medicine, Taipei Medical University, Taipei, Taiwan; ^2^ Department of Microbiology and Immunology, School of Medicine, College of Medicine, Taipei Medical University, Taipei, Taiwan; ^3^ Graduate Institute of Medical Sciences, College of Medicine, Taipei Medical University, Taipei, Taiwan; ^4^ Department of Microbiology & Immunology, Dalhousie University, Halifax, NS, Canada

**Keywords:** immunogenic cell death (ICD), oncolytic virus (OV), anticancer immunotherapy, pathogen-associated molecular patterns (PAMPs), damage-associated molecular patterns (DAMPs), viral engineering, combination therapy

## Abstract

According to the World Health Organization, cancer is one of the leading global health concerns, causing nearly 10 million deaths in 2020. While classical chemotherapeutics produce strong cytotoxicity on cancer cells, they carry limitations of drug resistance and off-target effects and sometimes fail to elicit adequate antitumor protection against tumor relapse. Additionally, most cancer cells have developed various ways to escape immune surveillance. Nevertheless, novel anticancer strategies such as oncolytic viro-immunotherapy can trigger immunogenic cell death (ICD), which can quickly grasp the attention of the host defense machinery, resulting in an ensuing antitumor immune response. Specifically, oncolytic viruses (OVs) can infect and destroy targeted cancer cells and stimulate the immune system by exposing pathogen-associated molecular patterns (PAMPs) and damage-associated molecular patterns (DAMPs) to promote inflammatory reactions, and concomitantly prime and induce antitumor immunity by the release of neoantigens from the damaged cancer cells. Thus, OVs can serve as a novel system to sensitize tumor cells for promising immunotherapies. This review discusses the concept of ICD in cancer, centralizing ICD-associated danger signals and their consequence in antitumor responses and ICD induced by OVs. We also shed light on the potential strategies to enhance the immunogenicity of OVs, including the use of genetically modified OVs and their combination with ICD-enhancing agents, which are helpful as forthcoming anticancer regimens.

## 1 Introduction

Cancers are the derivative of the genetic and epigenetic transformations leading to cell immortality and the generation of neoantigens. Neoantigens are newly formed proteins sourced by the mutated tumor-specific genes or tumor-associated viruses integrated into the genome, which previously have not been recognized by the host immune machinery (1). Furthermore, previous studies illustrated that the antitumor immune response provoked by tumor cell death could lead to prolonged therapeutic effects (2). The induced antitumor immunity is attributed to the alarming cellular molecules released by the dying cells in the tumor microenvironment (TME), and such danger cues are broadly referred to as “damage-associated molecular patterns” (DAMPs) (2, 3). Collectively, the immunogenicity conferred by tumor cell death depends on both antigenicity induced by neoantigen epitopes and adjuvanticity produced by specific DAMPs (4). This recipe for cell demise is recognized as “immunogenic cell death” (ICD).

Since the birth of the concept of ICD, the mode of viewing cancer therapy has drastically deviated in a different direction. ICD gained recognition as a host immunogenicity enhancer and initiator of lasting tumor-specific response as it recruits dendritic cells (DCs) and DC precursors. For successful immunogenicity, the danger signals’ production and exposure are critical for mediating cellular stress. However, cancer cells have designed various strategies to abrogate danger signals and dodge immune surveillance through loss of antigenicity or immunogenicity and *via* creating an immunosuppressive TME. Nonetheless, such immune evasion mechanisms mediated by cancer cells can be reversed by the evolving ICD inducers. Anticancer treatment strategies such as certain chemotherapeutics (e.g. the anthracycline family, oxaliplatin, and bortezomib) (5), radiotherapy (6), and photodynamic therapy (7) have been demonstrated to trigger ICD. A new category of ICD inducers—OVs can induce ICD specifically in targeted tumors. Microbial components from OVs can serve as pathogen-associated molecular patterns (PAMPs), which specific receptors can recognize in immune cells (8). Consequently, the discovery of OVs helped approach the viruses with a distinct perspective rather than viewing them as infectious disease-causing agents. With the advent of recombinant viruses, it is possible to engineer and develop attenuated viruses that retain their oncolytic ability while improving their tumor specificity (9, 10). Furthermore, the OVs combinatory approach with potent therapeutics has led to synergistic augmentations in ICD-induction and antitumor responses in several cancer types *in vitro* and *in vivo*. This review sheds light on the concept of ICD and summarizes the rationale and insights on preclinical research that led to the clinical trials of ICD-inducing OVs in cancer treatment.

## 2 Canonical mechanism of ICD: danger signaling and its consequence

During the ICD, dying cells secrete and express a broad panel of signature molecules on the cell surface in a defined temporal sequence (3). Recognition of these molecules, i.e., ICD-associated DAMPs, by specific pattern recognition receptors (PRRs) expressed on DCs ultimately results in the activation of both innate and adaptive immune responses (11).

### 2.1 Calreticulin

Calreticulin (CRT) is one of the early DAMPs that translocate from the ER to cell surface (ecto-CRT) during the pre-apoptotic stage by complexing with the disulfide isomerase ERp57 (12). Ecto-CRT facilitates the phagocytosis of antigens by DCs, thus triggering immune responses (13). The receptor for CRT is ambiguous; however, studies show it might act on CD91, a low-density lipoprotein receptor-related protein 1 (LRP1), scavenger receptors SREC-1 and SR-A, CD40 ligand, TRAIL, and Fas ligand present on innate immune cells, such as APCs (14–17). On the other hand, the phagocytosis mediating ability of CRT is counterbalanced by the robust expression of CD47, a CRT antagonist which serves as a ‘do not eat me signal’ (18). Besides, reconstituting CRT-deficient cancer cells with exogenous recombinant CRT could revive their susceptibility to phagocyte clearance and reverse their resistance to ICD inducers (19). Furthermore, in non-small cell lung cancer patients, neoplastic cells manifesting high CRT levels showed a favorable prognosis and enhanced accumulation of immune cells (20). Therefore, CRT is an essential antitumor immune booster as it plays a vital part in promoting a tumor-specific immune response.

### 2.2 Adenosine-triphosphate

The release of ATP from dying cells is one of the major hallmarks of ICD. Multiple pathways are required for ICD-associated ATP secretion, including autophagy, lysosomal exocytosis, apoptosis, membrane blebbing, and plasma membrane permeabilization (21). ATP released from apoptotic cells binds to the P2Y_2_ purinergic receptor on monocytes and macrophages to facilitate clearance of apoptotic cells (22). The released ATP also binds to the P2X_7_ purinergic receptor on DCs, triggering NALP3-ASC-inflammasome formation and discharge of IL-1β, which is essential for T cell priming (23). Of note, human breast cancer patients with the *P2RX7* loss-of-function allele were shown to have shorter metastatic disease-free survival (23), suggesting the important role of ATP secretion in ICD induction.

### 2.3 High mobility group box 1

When cells are undergoing ICD, high mobility group box 1 (HMGB1) is freed from the nucleus and translocated to the extracellular space through the permeabilization of both the nuclear lamina and the plasma membrane (11). The extracellular HMGB1 triggers DC maturation by activating TLR4 expressed on immature DCs. Once TLR recognizes HMGB1, it induces the transcription of the adaptor molecule myeloid differentiation response gene (MyD88). Reports suggest that the TLR4/MyD88 cascade inhibits lysosome and phagosome fusion which causes the phagocytic vesicle processing in DCs and expedites the uptake of tumor antigens by DCs (24–27). HMGB1, in its redox state, can elicit diverse immunomodulatory activities, such as a chemoattractant (reduced HMGB1) and a proinflammatory cytokine-mediator (oxidized HMGB1 possessing disulfide bond) (28, 29). However, some former studies proposed that HMBG1 may bind to T cell immunoglobulin and mucin domain-containing protein 3 (TIM-3) on DCs to mediate immunosuppressive function (30) or augment the immunosuppressive activities of myeloid-derived suppressor cells (MDSCs) (31). Therefore, its implication in cancer treatment is yet to be fully understood.

### 2.4 Heat shock proteins

Heat shock proteins (HSPs) are molecular chaperones generally located in the cytosol, ER, mitochondria, and nucleus and are crucial for their role in protein folding, refolding, and degeneration (32). Some HSPs including HSP70 and HSP90 were found to be released after cell death and act as alert stress signals to prime the immune cells (33). Extracellular HSPs serve as DAMPs recognized by the pattern recognition receptors (PPRs), such as TLR2 or TLR4, and stimulate APCs by enhanced expression of costimulatory molecules, MHC molecules, and proinflammatory and Th-1 cytokines (34, 35). In addition, HSPs can bind to distinct DC receptors and encourage the cross-presentation of the processed antigenic peptides. Typically, they act *via* scavenger receptors, activating intrinsic proinflammatory cascades like NF-kB, mitogen-activated protein kinases (MAPK/ERK), or associate with TLRs (36).

### 2.5 Annexin A1

Annexin A1 (ANXA1) belongs to the Ca^2+^-dependent phospholipid-binding protein family and plays a crucial part in resolving inflammatory responses (37). During ICD, secreted or surface-exposed ANXA1 can assist DC activity. Vacchelli et al. demonstrated that the interaction between ANXA1 and its receptor, formyl peptide receptor 1 (FPR1), on DCs is essential to direct DCs to the dying cancer cells. Tumors lacking *ANXA1* or hosts lacking FPR1 showed loss of immunotherapeutic outcome in anthracycline and oxaliplatin-treated mice (38). Besides, a loss-of-function polymorphic mutation in *FPR1* reduced the overall survival and progression-free survival in breast and colorectal cancer patients receiving anthracycline or oxaliplatin-based chemotherapy (38). These results suggested that ANXA1 in tumor cells and the presence of its receptor (FPR1) in hosts are essential to initiate antitumor T cell immunity.

### 2.6 Cellular nucleic acids, cytokines, chemokines, and translation factors

Cellular RNA, such as mRNA, released from damaged cells can promote the production of type I interferons (IFNs) and proinflammatory cytokines *via* the TLR3 signaling cascade (39). Therefore, uptake and recognition of extracellular self RNA in the endosomal compartment is crucial for the TLR3 signaling in tumor cells undergoing ICD (40). On the other hand, when recognized by cytosolic dsDNA sensor (CDS), dsDNA can mediate type I IFNs secretion *via* stimulator of interferon genes (STING) activation for an anticancer immune response (41). Type I IFNs aid in chemotherapy-induced ICD mainly through autocrine or paracrine signaling cascade, elicit immunostimulatory effect *via* IFNAR signaling in immune cells, and trigger CXCL10 secretion by tumor cells (40, 42). Notably, CXCL10 secreted due to anthracycline-induced ICD of tumor cells can recruit T cells to the TME (40). In addition, growing evidence supports that determining the phosphorylation levels of eukaryotic translation initiation factor 2α (eIF2α) can be a potential marker for auditing the ICD process, since eIF2α phosphorylation is mandatory for the pre-apoptotic surface exposure of ecto-CRT (43).

**Figure 1:**
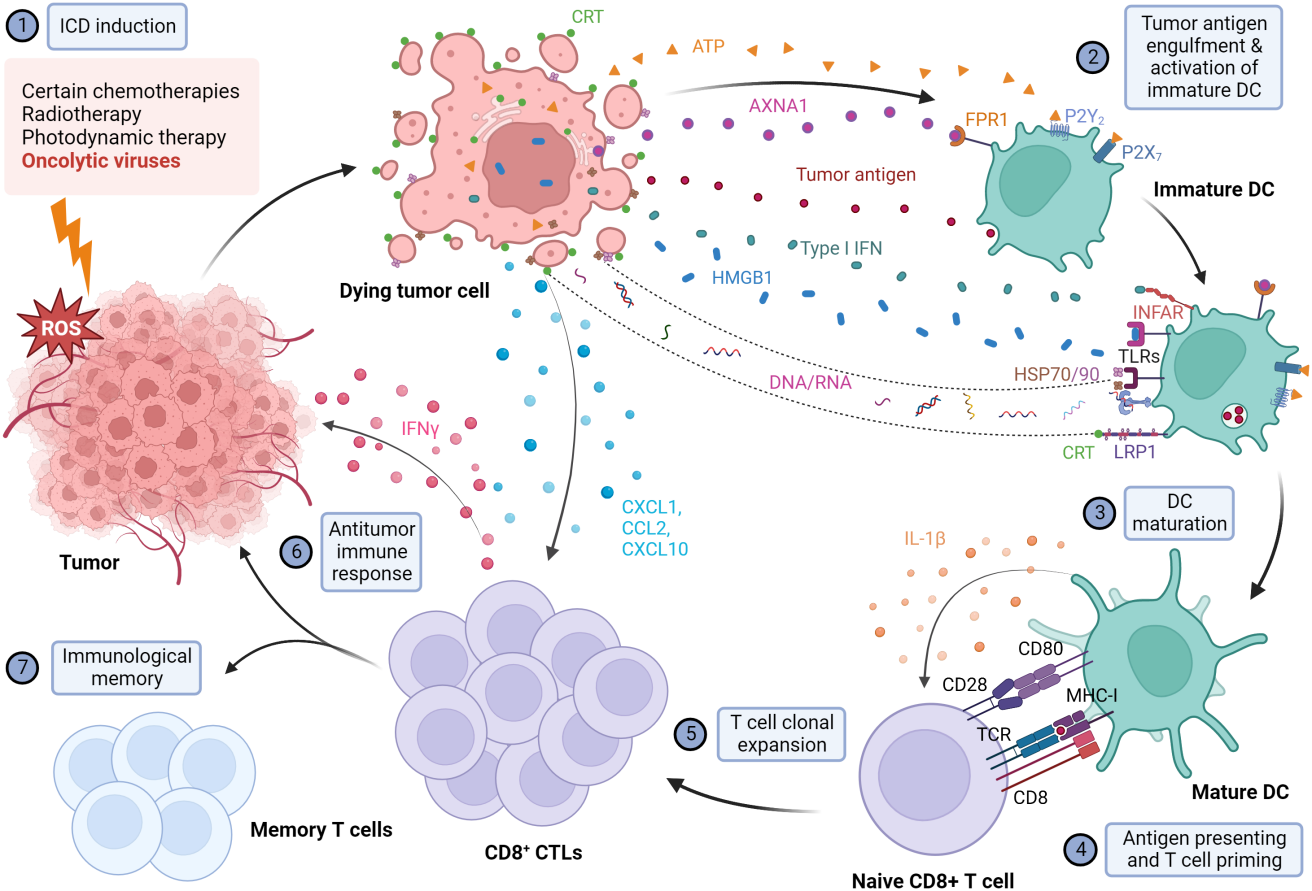
Illustration of the mechanism of immunogenic cell death and the subsequent activation of the antitumor immune response. When exposed to different immunogenic cell death (ICD) inducers, including OVs, cancer cells are under extreme ER stress and undergo ICD. Dying cancer cells express various damage-associated molecular patterns (DAMPs), including the release of high mobility group box 1 (HMGB1) from the nucleus, translocation and cell surface exposure of calreticulin (ecto-CRT) and heat shock proteins HSP70/90, and extracellular secretion of ATP, Annexin A1 (AXNA1), cytokines, chemokines, and nucleic acids. Exposure to DAMPs serves as a "find me" signal which recruits immature DC to TME and induces the maturation of DC. Ecto-CRT provides a pro-phagocytic signal that promotes the phagocytosis of antigens by DC. In addition, HMGB1 and HSP70/90 assist in promoting the processing of phagocytic cargo by binding to toll-like receptors (TLRs), thereby escalating antigen engulfment, processing, and presentation to T cells to mediate tumor-specific immune response and protective immunological memory. Created with BioRender.com.

Overall interactions between DAMPs with purinergic receptors, phagocytic receptors, and PRRs in host cells are crucial in stimulating DCs to actively display antigens to major histocompatibility complex (MHC) I/MHC II molecules on T cells for a tumor antigen-specific antitumor response. Therefore, immunogenic dying cells could mediate the phagocytic engulfing of dying tumor cells and target the leftover malignant cells (**Figure 1**).

## 3 Oncolytic viruses — an emerging anticancer modality over conventional chemotherapies

Traditional cytotoxic chemotherapeutic drugs can restrict the rapidly growing tumor cells by inducing DNA damage, disrupting DNA synthesis or repair, or targeting the basic functions of cell division (44) but can face significant obstacles when cancer stem cells develop drug resistance. A noticeable example is the expression of specific ATP-binding cassette (ABC) transport protein, also known as breast cancer resistance protein (BCRP)/ATP-binding cassette superfamily G member 2 (ABCG2) protein in hematopoietic stem cells and cancerous cells (45, 46). BCRP-ABCG2 protein effluxes the intracellular toxic-chemical agents to the extracellular space by utilizing the energy liberated by ATP hydrolysis (47). In acute myeloid leukemia (AML), BCRP-ABCG2 is predominantly expressed in stem cell subpopulations to mediate energy-driven clearance of toxic agents (48). Other drug resistance mechanisms may also be acquired, such as by the elevated expression of detoxifying enzyme aldehyde dehydrogenase (ALDH) against alkylating agents (e.g., cyclophosphamide) (49). Additionally, chemotherapy is often associated with patient toxicities including immunosuppression with reduced level of immune cells, increasing the chance of therapy intolerance (44).

On the contrary, OVs can selectively target and lyse cancer cells. Some OVs exhibit natural tropism in cancerous tissues, while some OVs are genetically modified to detect specific targets, replicate in cancer cells, and deliver certain genes. The unique feature of OVs is their ability to distinguish tumor cells from healthy cells, resulting in more tumor-specific toxicity, unlike conventional chemotherapy which flows to every region in the systemic circulation and damages the fast-growing normal cells. Above all, the OVs can activate the immune system against cancer cells to elicit potent antitumor responses. Several OVs can trigger ICD *via* the expression of danger signals (i.e., DAMPs), thereby marking the dying tumor cells for recognition by the innate immune cells. Apart from this, OVs elicit a specific antiviral immune response following ICD induction for virus clearance to prevent possible virus-induced toxicity. PRRs including toll-like receptors (TLRs), RIG-I-like receptors (RLRs) and cytoplasmic DNA receptors can recognize viral PAMPs, which activate the antiviral innate immune responses that induce the expression of the type I IFNs and pro-inflammatory cytokines (50). Type I IFNs can also trigger the adaptive immune response by priming T helper cells and cytotoxic T cells, leading to the induction of antigen-specific responses (50). In addition, OVs can serve as a vector to deliver the desired therapeutic agent to target cells. Several studies also demonstrated that combining OV with traditional therapies showed synergistic tumor killing, implying that OV’s tumor-specificity and antitumor feature may be a booster for adequate antitumor protection in combination therapy (51). The features above make it ideal to employ ICD-inducing OVs as a novel treatment alternative in cancer therapy.

## 4 Immunogenic and multimodal cell death mediated by OVs

**Figure 2:**
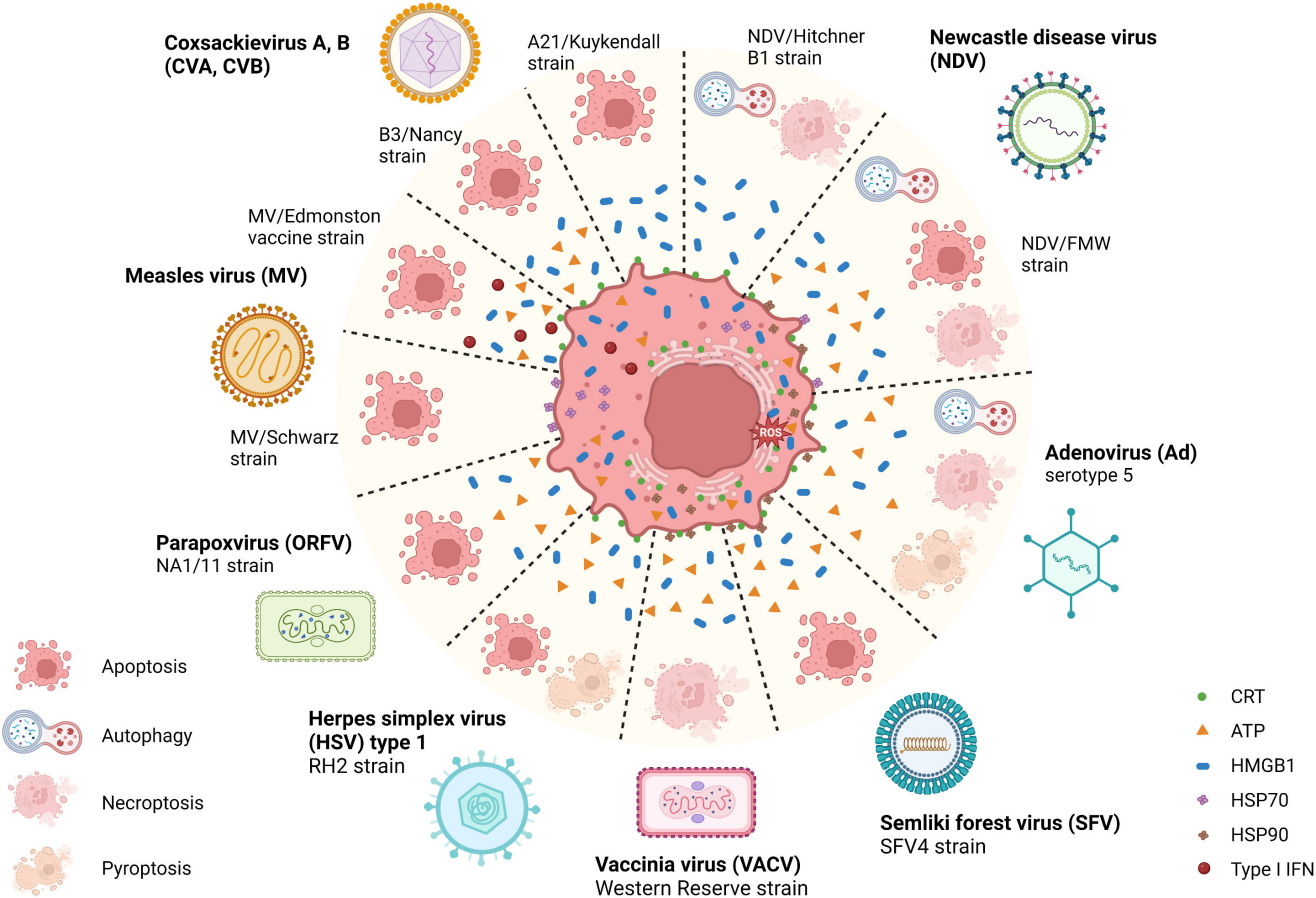
Illustration of the immunogenic cell death related pathways induced by natural OVs. Several natural oncolytic viruses (OVs), including coxsackievirus A, B (CVA, CVB), Newcastle disease virus (NDV), adenovirus (Ad), semliki forest virus (SFV), measles virus (MV), herpes simplex virus (HSV) and poxviruses, such as vaccinia virus (VACV) and parapoxvirus (ORFV) can induce a distinct mode of immunogenic cell death (ICD). For example, CVA, CVB, MV, ORFV, and SFV induce immunogenic apoptosis, and VACV induces necroptosis. While some OVs, such as HSV, NDV, and Ad, induce multimodal cell death, mediating chronic exposure of DAMPs in dying cancer cells. During this phase, the dying tumor cells exposing multiple damage-associated molecular patterns (DAMPs) recruit innate immune responders, thus connecting the bridge between TME and host innate immune machinery, eliciting a potent antitumor immune response. Created with BioRender.com.

OVs can induce ICD with various features, such as causing ER stress, HMGB1 and ATP release, and elevated surface expression of CRT during infection. A recent study illustrated that parapoxvirus *ovis* (ORFV) induced extracellular secretion of HMGB1 and ATP in lung cancer cells (52). Squamous cell carcinoma (SCC) cells infected with herpes simplex virus type 1 (HSV-1) manifested extracellular ATP and HMGB1 release, as well as CRT translocation to the membrane (53). Similarly, Adenovirus (Ad), Semliki Forest virus (SFV4), and Vaccinia virus (VACV) mediated CRT exposure, the release of HMGB1 and HSP90, as well as ATP release in lung cancer and bone cancer cells (54). The attenuated poliovirus (PV)-based vector PVSRIPO (55, 56), which has its Internal Ribosomal Entry Site (IRES) substituted with IRES from rhinovirus type 2 (HRV2) to eliminate PV’s neurovirulence, was shown to induce HSPs, HMGB1, viral dsRNA, and tumor antigen release from melanoma and breast cancer cells (57). Besides, some related OVs may induce differential ICD hallmarks in distinct tumors. For example, Coxsackievirus B3 (CVB3) mediated cytotoxicity in lung cancer cells with high CRT exposure, HMGB1 release, and ATP secretion (58), whereas CVA21 mediated cytotoxicity in bladder cancer cells positively correlated with high CRT exposure and HMGB1 release but not ATP release (59). In parallel, the Newcastle disease virus (NDV) Hitchner B1 strain induced surface translocation of CRT and HMGB1 secretion in glioma cells, which produced a long-term, tumor-specific immunological memory response in an orthotopic glioma model (60). NDV/FMV strain-infected melanoma, lung cancer, and prostate cancer cells also displayed HSP70/90 and ATP secretion in addition to CRT exposure and HMGB1 release (61–63). As for measles virus (MV), the Edmonston strain mediated the release of type I IFNs and HMGB1 in human melanoma (64) and CRT exposure, ATP, and HMGB1 release in hepatocellular carcinoma (HCC) (65). MV-Schwarz strain mediated the release of HSP70 in mesothelioma cells (66). Examples of ICD-inducing OVs are summarized in **Figure 2** and **Table 1**.

**Table 1:** ICD hallmarks induced by OVs.

ICD inducer	Cancer type	Cell death process	ICD markers	Outcome(s)	Refs
***Natural OVs***					
Coxsackievirus A21 (CVA21) Kuykendall prototype strain	Bladder carcinoma	Apoptosis	Ecto-CRT, HMGB1	Prophylactic protection, tumor-specific CD4^+^ T cell immune response *in vivo*	(59)
CVB3 strain Nancy	Lung adenocarcinoma	Apoptosis	Ecto-CRT, HMGB1, ATP	Significant aggregation of NK cells, granulocytes, macrophages, and DCs into tumor tissues, complete tumor destruction, and extended survival benefits *in vivo*	(58)
Newcastle disease virus (NDV) strain Hitchner B1	Glioma	Necroptosis, autophagy	Ecto-CRT, HMGB1	Elevated IFN-γ ^+^ T cells infiltration, decreased aggregation of MDSCs, and tumor-specific immunological memory *in vivo*	(60)
NDV strain FMW (NDV/FMW)	Lung carcinoma	Autophagy	Ecto-CRT, HMGB1, HSP70/90	Significant inhibition of tumor progression *in vivo*	(62)
NDV/FMW	Prostate adenocarcinoma	Apoptosis	Ecto-CRT, HMGB1, HSP70/90	Significant inhibition of tumor progression *in vivo*	(63)
NDV/FMW	Melanoma	Apoptosis, autophagy, necroptosis, ER stress	Ecto-CRT, HMGB1, HSP70/90, ATP	Significant inhibition of tumor progression *in vivo*	(61)
Adenovirus (Ad)-serotype 5	Lung carcinoma	Autophagy, necroptosis, pyroptosis	Ecto-CRT, HMGB1, HSP90, ATP	Engulfment of virus-infected tumor cells by DCs, secretion of Th1 cytokines by DCs, and tumor-specific T cell immune response *in vitro*	(54)
Semliki forest virus (SFV) strain SFV4	Osteosarcoma, lung adenocarcinoma	Apoptosis	Ecto-CRT, HMGB1, HSP90, ATP	DC-ingestion of virus-infected tumor cells, secretion of Th1 cytokines by DCs, and tumor-specific T cell immune response *in vitro*	(54)
Vaccinia virus (VACV) Western Reserve strain	Osteosarcoma, lung adenocarcinoma	Necroptosis	Ecto-CRT, HMGB1, HSP90, ATP	DC-ingestion of virus-infected tumor cells, suppression of cytokine secretion by DCs, and counteraction of immunogenicity *in vitro*	(54)
Herpes simplex virus (HSV) type 1 RH2 strain	Squamous cell carcinoma	Apoptosis, pyroptosis	Ecto-CRT, HMGB1, ATP	Significant reduction of tumor volume *in vivo*	(53)
Parapoxvirus (ORFV) strain NA1/11	Lung carcinoma	Apoptosis	HMGB1, ATP	Significant reduction of tumor volume, augmentation of CD80 and CD86 on CD11c^+^ DCs, increased levels of CD8^+^ Granzyme B^+^ T cells, and CXCL16 mediated migration of CD8^+^ T cells *in vivo*	(52)
Measles virus (MV) Edmonston vaccine strain (Edm)	Melanoma	Apoptosis	HMGB1, Type I IFN	Upregulation of CD69 NK cell activation marker, augmentation of CD80 and CD86 on DCs, Th1 cytokine response, and specific CD8^+^ T cell response *in vitro*	(64)
MV-Edm	Hepatocellular carcinoma	Apoptosis	Ecto-CRT, ATP, HMGB1	Promote CD8+ NKG2D+-mediated oncolysis *in vitro*; Significant inhibition of tumor growth, suppression of metastasis, prolonged survival *in vivo*	(65)
MV-Schwarz strain	Mesothelioma	Apoptosis	HSP70, dsRNA	Activation of autologous antitumor response *in vitro*	(66)
***Modified OVs***					
Ad5/3-hTERT-E1A-hCD40L	Lung adenocarcinoma, bladder carcinoma	Apoptosis	Ecto-CRT, HMGB1, ATP	Significant inhibition of tumor growth, Th1 response, CD8^+^ T cells activation, and declined immunosuppression *in vivo*	(67)
Ad/OBP-702-p53	Pancreatic adenocarcinoma	Apoptosis, autophagy	HMGB1, ATP	Significant suppression of tumor growth and recruitment of tumor antigen-specific CD8^+^ T cells *in vivo*	(68)
Telomelysin (OBP-301)	Colon carcinoma, pancreatic ductal adenocarcinoma	Apoptosis, autophagy	HMGB1, ATP	Recruitment of tumor antigen-specific CD8^+^ T cells, inhibition of Foxp3^+^ T cell infiltration, suppression of both primary and metastatic tumors *in vivo*	(69)
Fusogenic VACV (FUVAC)	Lung carcinoma, colon carcinoma	Apoptosis, necrosis	HMGB1, ATP	Increased infiltration of tumor-specific CD8^+^ T cells, inhibition of tumor-associated immune suppressive cells, suppression of both primary and metastatic tumors, and tumor-specific immunological memory *in vivo*	(70)
VACV/MVA-TAA-4-1BBL	Melanoma, colon carcinoma	–	HMGB1	Reactivation and expansion of CD8^+^ T cells, the release of proinflammatory molecules, suppressed local and distant tumor relapse, and tumor-specific immune memory *in vivo*	(71)
VACV/WR strain (VVWR/TK^-^RR^-^/FCU1)	Fibrosarcoma, colon carcinoma, melanoma	Autophagy, apoptosis	HMGB1, ATP, ecto-CRT, CXCL10	Abscopal effect on distant tumors and activation of CD8^+^ T cell response *in vivo*	(72)
NDV-MIP3α	Melanoma, colon carcinoma	Apoptosis	HMGB1, ATP	Significant suppression of tumor growth, tumor-specific cellular, and humoral immunity *in vivo*	(73)
Talimogene laherparepvec (T-VEC)/HSV-1-GM-CSF	Melanoma	–	Ecto-CRT, HMGB1, ATP	Recruitment of tumor-specific CD8^+^ T cells, antiviral immune response, and induction of proinflammatory gene signatures *in vivo*.	(74)
T-VEC	Melanoma	–	Ecto-CRT ATP	Delayed yet significant reduction of tumor cell viability, maturation of human BDCA-1^+^ myDCs *in vitro*	(75)
PVSRIPO	Melanoma, prostate cancer, breast cancer	–	HMGB1, HSPs 60/70/90, dsRNA, p- eIF2α	DCs and macrophages type I IFN-dominant activation, innate anti-pathogen inflammatory response, and tumor-specific immune response *in vitro*; delayed tumor growth, improved survival, and antitumor immunity *in vivo*	(57)

ICD, immunogenic cell death; Ecto-CRT, surface-exposed calreticulin; HMGB1, high mobility group box 1; ATP, adenosine-tri-phosphate; NK cells, natural killer cells; DCs, dendritic cells; IFN, interferon; MDSCs, myeloid derived suppressor cells; HSP60/70/90, heat shock protein 60/70/90; ER, endoplasmic reticulum; Th1, T helper type 1 cells; CD 11c/69/80/86, cluster of differentiation 69/80/86; CXCL10/16, C-X-C motif chemokine ligand 10/16; NKG2D, natural-killer group 2, member D receptor; dsRNA, double-stranded RNA; Foxp3, forkhead box P3; BDCA-1^+^ myDCs, blood dendritic cell antigen 1-positive myeloid DCs; p-eIF2α, phosphorylated eukaryotic translation initiation factor 2α.

The fate of tumor cells undergoing ICD establishes tumor-specific immune effects by cross-priming tumor antigens to T cells *via* APCs. For instance, the innate immune cells are recruited in TME to uptake, process, and present tumor-specific antigens in human non-small cell lung cancer (58). For the application in oncolytic viro-immunotherapy, OVs must cause the proper ways of cell death that elicit robust antitumor immunity. Initial investigations of ICD were associated only with the concept of “immunogenic apoptosis,” but over a decade of research shed light on multimodal cell death related pathways, including necrosis, necroptosis, pyroptosis, and autophagy (76). Various tumors and associated endothelial cells killed by OVs showed distinct types of cell death, characterized by the drastic fine-structural transition of dying cells. Predominantly, immunogenic apoptosis is the most common mode of cell death induced by OVs. SFV4 replication displayed rapid cell lysis coupled with induction of an intrinsic caspase-3/7-mediated apoptotic pathway (54). CVA21, CVB3, and ORFV induced immunogenic apoptosis of bladder cancer cells and lung cancer cells, respectively, while ORFV enhanced DC activation in infected cancer cells (52, 58, 59). In addition, MV-infected melanoma, HCC and mesothelioma cells, and NDV-infected prostate cancer cells were also susceptible to immunogenic apoptosis (63–66).

Nonetheless, many studies have emphasized the significance of necroptosis, a programmed necrotic cell death process in a caspase-independent fashion, in overcoming apoptotic resistance and may induce or augment antitumor immunity. Many key mediators in necroptotic signaling cascades are less expressed in various tumors, implying that malignant cells may dodge necroptotic hook for survival (77, 78). For example, VACV infection in lung cancer cells showed decreased caspase-8 activity with a marked increase in phosphorylated mixed lineage kinase domain-like (MLKL), a known obligate effector of necroptosis (54). Some OVs also trigger multiple cell death processes in the target cancer cells. For example, wild-type Ad-serotype 5 induced autophagy and activated necroptotic and pyroptotic cell death pathways (54). The immunogenic killing of HSV-1 in SCC was reduced by pan-caspase and caspase-1 inhibitors, demonstrating the involvement of apoptosis and pyroptosis (53). In the case of NDV, NDV/Hitchner B1 was suggested to induce necroptosis rather than apoptosis in glioma cells (60), whereas NDV/FMW induced autophagy-dependent ICD (not affected by apoptosis and necroptosis inhibitors) in lung cancer cells (62) and multiple cell death processes including autophagy, apoptosis, and necroptosis in melanoma cells (61). These results suggest that OVs may trigger various cell death modalities in cancer cells, and the types of cell death induced may depend not only on the virus but also on the cancer type.

## 5 Strategies to modulate the immunogenic efficacy of OVs

Although OVs have selective tumor lysis potential, antiviral immunity may restrict OVs’ replication and spread, leading to premature OV clearance (79). In addition, numerous preclinical and clinical research investigated the antitumor efficacy of “non-armed” OVs; however, their immune-provoking ability does not always produce the anticipated antitumor response. Therefore, strategies to modulate the immunogenicity of OVs may be beneficial to enhance their therapeutic potential in cancer immunotherapy.

### 5.1 Genetic engineering of OVs to improve oncolysis, innate and adaptive immune responses

Viruses can be engineered at the gene level to optimize antitumor immunity by delivering various immuno-modulatory transgenes, most prominently DC and T cell activating cytokines (80).

#### 5.1.1 Cell death-inducing factors for enhanced oncolysis

Researchers have introduced cell death-inducing factors in OV vectors for more efficient immune-oncolytic therapeutic outcomes. For example, Van Hoecke et al. found that intratumoral delivery of mRNA encoding MLKL, a necroptosis-inducing factor, induced tumor lysis and mediated antitumor immunity in mouse models (81). In a follow-up study, they employed wild-type VACV for targeted intratumoral delivery of MLKL, which led to the activation of necroptosis-like tumor cell death *in vitro* (82). Furthermore, MLKL expressing VACV vectors induced a noticeable antitumor activity coupled with potent intrinsic antitumor immunity against neo-epitopes (82). On the other hand, Ad OBP-702 expressing p53, a tumor suppressor protein, substantially enhanced ICD *via* HMGB1 and ATP release by favoring apoptotic and autophagic cancer cell death (68). Ad/OBP-702-p53 thus promoted significant CD8^+^ T cell infiltration in pancreatic ductal adenocarcinoma (PDAC) tumors to achieve efficient antitumor response (68).

#### 5.1.2 Immuno-modulatory transgenes to optimize APCs activation

The first U.S. FDA-approved oncolytic virus, talimogene laherparepvec (T-VEC), is an attenuated HSV-1 armed with two copies of human granulocyte-macrophage colony stimulating factor (GM-CSF) gene to promote DC infiltration and maturation (74). T-VEC was shown to induce apoptosis in melanoma cells (83) and trigger ICD hallmarks including ecto-CRT, HMGB1, and ATP release (74, 75), resulting in the recruitment of tumor-specific CD8+ T cells and induction of proinflammatory responses in both injected and non-injected tumor sites (74). On the other hand, expressing CD40 ligand (CD40L) to the CD40 receptor on APCs leads to cancer cell apoptosis and Th-1 immune reaction, followed by cytotoxic T cell activation and nullified immunosuppression. Chimeric Ad5/3-hTERT-E1A-hCD40L, coding for CD40L, substantially reduced tumor development by oncolytic and apoptotic properties *in vivo* (67). (Ad5/3-hTERT-E1A-hCD40L)-induced oncolysis culminated in ATP and HMGB1 release and augmented CRT exposure implying immunogenicity. *In vivo* therapeutic intervention of immunocompetent mice with Ad5/3-hTERT-E1A-hCD40L showed macrophages’ recruitment and activation, which induced Th-1 cell immune response *via* IL-2 release. This effect was coupled with a cytotoxic T cell response *via* CCL5/RANTES, IFN-γ, and TNF-α cytokines, suggesting the activation of a systemic T cell immune response (67). Recombinant NDV NDV-MIP3α expressing the macrophage inflammatory protein-3α (MIP3α), a chemokine of DCs, exhibited tumor killing and ICD initiation, same as the wild-type NDV *via* HMGB1 and ATP release (73). Moreover, NDV-MIP3α successfully promoted the maturation and activation of DCs with strong expression of CD80 and CD86 costimulatory markers and IFN-γ and TNF-α active cytokines. Ultimately, significant reversion of TME and tumor-specific cellular and humoral responses are observed in melanoma and colorectal murine models (73).

#### 5.1.3 Immuno-modulatory transgenes to optimize T cell immune responses

T cell activation demands a minimum of two signals, including a TAA-specific signal dispatched *via* a T cell receptor and a costimulatory signal attributed *via* designated molecules. Previous studies have shown that intratumoral delivery of immunomodulatory genes such as B7-1 and B7-2 could augment antitumor responses (84, 85). The inoculation of recombinant VACV expressing the murine B7-1 or B7-2 genes (rVACV-B7-1 or rVACV-B7-2) into immunocompetent mouse models appeared to halt the tumor growth (84). Similarly, Todo et al. developed a defective HSV-1 vector incorporated with B7-1-immunoglobulin (B7-1-Ig) fusion transgene (dvB7Ig/G207) (85). Intraneoplastic inoculation of dvB7Ig/G207 in the neuroblastoma syngeneic mouse model inhibited the cancer progression and substantially increased CD4^+^ and CD8^+^ T cell infiltration, and the mice cured by dvB7Ig/G207 treatment were protected against tumor rechallenge (85). Furthermore, lack of adequate stimulation and sufficient tumor infiltration due to immune-hostile TME often downgrades the naturally primed CD8+ T cell response. However, intratumoral delivery of the VACV/MVA vaccine strain armed with 4-1BBL (a T cell immunostimulatory molecule) led to reactivation and increased expansion of tumor-specific CD8^+^ T cells (71). In addition, MVA-TAA-4-IBBL induced strong ICD with HMGB1 exposure and generated tumor-specific immune memory that eliminated local and distant tumor relapse (71).

#### 5.1.4 Recombination of OVs to enhance immunogenicity

Recombination of OVs has been explored to enhance the oncolytic activity and immunogenicity of viral vectors. For example, the reovirus p14 fusion-associated small transmembrane (FAST) protein, a viral fusogen that induces cell-cell fusion and syncytia formation, has been recombined with the attenuated vesicular stomatitis virus vector VSVΔM51 to form the VSV-p14 chimera (86). VSV-p14 infected breast cancer spheroids more efficiently and enhanced immune cell activation in syngeneic breast cancer mouse model compared to VSVΔM51 encoding green fluorescence protein (VSV-GFP) (86). Likewise, recombinant VSV-NDV (rVSV-NDV) chimera is produced by retaining the highly replicative VSV backbone and replacing its glycoprotein with the hemagglutinin-neuraminidase (HN) and modified fusion (F) envelope protein of fusogenic NDV to improve the efficacy and safety of VSV (87). rVSV-NDV induced tumor-specific syncytia formation, followed by dynamic cell-to-cell virus transmission for rapid onset of ICD in HCC, as observed by ATP, HMGB1, Hsp70, and Hsp90 release and CRT expression (87). Systemic administration of rVSV-NDV substantially increased the overall survival of the orthotopic HCC-bearing mice (87). Therefore, combining the valuable traits of two or more OVs may pave an attractive and beneficial platform for the clinical safety and efficacy of oncolytic viro-immunotherapy.

### 5.2 Combination of OVs and ICD-enhancing agents for enhanced immunotherapeutic efficacies

This approach uses OVs with ICD-enhancing chemical drugs, some of which are clinically available, to break immune tolerance and induce a long-lasting tumor-specific immune response.

#### 5.2.1 Enzyme inhibitors

Bortezomib is a novel peptide-based proteasome inhibitor. The ICD-inducing property comes from its ability to cause reactive oxygen species (ROS) generation and mitochondrial dysfunction, which leads to cellular stress and DAMP release (88, 89). In HCC, ICD induced by Ad expressing human telomerase reverse-transcriptase (hTert-Ad) infection is enhanced with bortezomib treatment. Proteasome inhibition during hTert-Ad infection triggered the complementary ER stress pathways by negatively disrupting unfolded protein response (UPR) and augmented the caspase-dependent activation of antitumor immunity (90). Furthermore, bortezomib repressed the antiviral immune response in immunocompetent mice, which improved oncolysis of hTert-Ad (90). In the immunocompetent HCC set-up, adenovirotherapy-induced CD8^+^ T cell immunity suppressed the primary tumor and the offshoot of non-infected lung metastases (90). In another study, VSV and bortezomib showed an antagonistic effect against myeloma cells *in vitro*, but in contrast, a synergistic anti-myeloma effect was observed *in vivo*. This effect is explained by the bystander immune cells in host TME absent *in vitro* (91), suggesting the possible ER stress-induced ICD upon proteasome inhibition.

#### 5.2.2 STAT3 inhibitors

Several signaling pathways are aberrantly hyperactivated in diverse cancers, among which activation of transcription factor STAT3 accounts for immunosuppression. Hence, hindering STAT3 may provide new strategies for anticancer immunotherapies. Wang et al. demonstrated that impairing STAT3 signaling cascade with small-molecule STAT3 inhibitor, C188-9, which binds to the phosphotyrosyl peptide binding site within STAT3, has significantly reduced the tyrosine phosphorylation status of STAT3 in prostate cancer cells (63). This C188-9 pre-treatment, followed by NDV/FMW infection, profoundly enhanced HMGB1 and HSP70/90 release and increased CRT exposure. To eliminate possible off-target effects of C188-9, stable knock-down of STAT3 with lentivirus-based shRNA has significantly elevated NDV/FMW-induced tumor growth inhibition, ICD, and substantially augmented all three ICD hallmarks as in C188-9 pre-treatment (63). Furthermore, the supernatant from NDV/FMV-infected prostate cancer cells significantly repressed tumor growth *in vivo* (63). On the contrary, the same group showed that STAT3 is crucial in the NDV/FMW-caused ICD process in melanoma cells (61). Pharmacological inhibition or shRNA-mediated depletion of STAT3 suppressed the NDV/FMW-caused ICD determinants, including HMGB1 and HSP70/90 release, CRT exposure, and ATP secretion (61), suggesting plausible mechanisms to fine-tune NDV-induced ICD in diverse cancer types.

#### 5.2.3 Immune checkpoint inhibitors

A class of cancer immunotherapy, immune checkpoint inhibitors (ICIs), has recently caused a paradigm deviation that dramatically enhanced the clinical output in malignant tumor therapies, such as gastric cancer, melanoma, Hodgkin’s lymphoma, and non-small cell lung cancer (92–95). Among these, blocking antibodies against programmed cell death-1 (PD-1) combined with potential OVs synergized the OVs-induced ICD, thus favoring the combination therapy. A telomerase-specific oncolytic Ad (Telomelysin, OBP-502) caused the release of ICD determinants, such as ATP and HMGB1, and chemokines such as RANTES and CXCL10/IP-1, resulting in CD8^+^ T cell recruitment and blockade of intratumoral penetration of Foxp3^+^ T cells (69). Combining intratumoral OBP-502 administration and anti-PD-1 Ab systemic administration in bilateral subcutaneous tumor models has elicited an abscopal effect by gradually repressing both OBP-502-treated tumors and OBP-502-non-treated tumors by active recruitment of CD8^+^ T cells (69). In addition, the combination efficacies are evaluated in a more clinically relatable orthotopic rectal tumor model with liver metastasis, which significantly prolonged survival and gravely suppressed both primary and metastatic tumors (69). Moreover, a novel fusogenic oncolytic vaccinia virus (FUVAC) displayed fusogenic cytopathic effects resulting in cell-cell fusion, apoptosis, necrosis, and ICD *via* HMGB1 and ATP release, which increased CD8^+^ T cell infiltration and decreased tumor-associated immune repressive cells (70). Strikingly, combining FUVAC and anti-PD-1 Ab offered synergistic effects by achieving complete response (CR), abscopal effect against non-treated distant tumors, and antitumor immunological memory against tumor re-implantation (70). Most importantly, coupling ICD-inducing OVs with anti-PD-1 monotherapy may have positive outcomes in areas of significant unmet needs. For instance, the VACV/GLV-1H68 strain induced ICD with the substantial secretion of ATP, HMGB1, and surface exposure of CRT (96). Direct delivery of VACV/GLV-1H68 using isolated limb perfusion (ILP) and subsequent PD-1 blockade showed no local and distant relapse with significant survival in neoadjuvant models (96). Likewise, VACV/WR strain (VVWR/TK^-^RR^-^/FCU1) mediated an abscopal effect on distant tumors by activating CD8^+^ T cell response (72). This systemic efficacy of VACV/WR was further augmented when combined with anti-CTLA-4 and anti-PD-1 ICIs, thereby displaying a higher survival benefit (72).

#### 5.2.4 Vitamins

Novel insights into the pharmacological action of vitamin C (Vit-C) suggest that high-dose Vit-C can trigger the accumulation of large quantities of ROS through multiple pathways, which ultimately damages cancer cells (97). High-dose Vit-C was also shown to enhance ICIs (97). Recently, Ma et al. demonstrated that combination therapy of high-dose Vit-C and oncolytic Ad could mediate synergistic antitumor effect by augmenting ICD (98). This combination resulted in significant infiltration of T cells in TME, activation of CD8^+^ T cell-dependent antitumor effects, and decreased proportion of regulatory T cells (98). Hence, OVs and Vit-C combination regimens may offer new dimensions in cancer immunotherapy and deserves further exploration.

## 6 Conclusions and prospects

Cancers are known to hijack routine cell regulatory cascades, escape immune surveillance, and adapt to various anticancer treatment strategies, thereby diminishing therapeutic efficacies. Nonetheless, OVs identified for their abilities to selectively kill cancer cells and induce systemic antitumor immune response through ICD are arising as a new class of potent anticancer agents. Furthermore, recent progress has demonstrated that genetic engineering of OVs with transgenes and combining OVs with ICD-enhancing agents can potentiate ICD and establish a robust antitumor response with immunological memory. These aspects underscore ICD as a key player and essential cornerstone of oncolytic viro-immunotherapy. Future development of advanced OV regimens will need to consider how to achieve optimal and balanced oncolytic activity in inducing ICD, promoting tumor clearance, and eliciting effective long-lasting antitumor protection, which are vital for a successful oncolytic viro-immunotherapy as a next-generation anticancer modality.

## Author contributions

Conceptualization: LP, C-HL, and L-TL. Writing—Original Draft: LP and C-HL. Writing—Review and Editing: LP, C-HL, and L-TL. Supervision: L-TL. Funding Acquisition: L-TL. All authors contributed to the article and approved the submitted version.
